# The Bacillus subtilis putative LysR-type transcriptional regulator YybE and its connection to chromosome replication and segregation

**DOI:** 10.1099/acmi.0.001000.v4

**Published:** 2026-01-07

**Authors:** Alan Koh

**Affiliations:** 1 Centre for Bacterial Cell Biology, Biosciences Institute, Faculty of Medical Sciences, Newcastle University, Newcastle upon Tyne, UK

**Keywords:** *Bacillus subtilis*, chromosome morphology, DNA replication initiation, LysR-transcriptional regulator, YabA, YybE

## Abstract

Duplication and segregation of genetic material are vital for cell proliferation. Deletion of DNA replication regulators, such as YabA and ParA, leads to over-initiation of DNA replication. However, the viability of the *ΔyabA ΔparA* double mutant suggests additional regulatory mechanisms. Using a transposon mutagenesis library, *yybE* was identified as a potential candidate. Bioinformatic analysis of *yybE* suggests that it encodes a putative LysR-type transcriptional regulator (LTTR). LTTRs are established regulators of metabolic processes, leading to the hypothesis that YybE might link metabolic processes to DNA replication. However, under the tested conditions, deletion of *yybE* did not result in detectable changes to DNA replication frequency, origin segregation or chromosome morphology.

## Data Summary

Analysed data are available through Figshare, https://doi.org/10.6084/m9.figshare.28360835.v2. The author confirms that all supporting data and protocols have been provided within the article or through supplementary data files.

## Introduction

DNA replication initiation must be precisely regulated to ensure the accurate duplication and segregation of the genome during cell division. Disruption in this process leads to genomic instability, which can compromise cell viability. In bacteria, multiple overlapping regulatory mechanisms safeguard DNA replication initiation, reflecting its critical importance to cellular fitness. One such potential regulator is the LysR-type transcriptional regulator (LTTR) [1,2]. They represent a diverse family of bacterial transcriptional regulators and are typically DNA-binding proteins that are involved in a range of cellular processes by modulating bacterial responses to environmental or intracellular cues [1,6]. Bacterial metabolic states and nutrient availability are factors that are known to influence DNA replication states [7].

Additionally, another layer of bacterial regulatory mechanism is the proteins that directly regulate DNA replication initiation. Protein such as YabA, a conserved protein in many Gram-positive bacteria, plays a role in preventing over-initiation of DNA replication [8,9]. YabA is thought to form a molecular bridge between the replication initiator protein DnaA and the sliding clamp DnaN, effectively sequestering DnaA from *oriC* to inhibit reinitiation [9,14]. While deletion of *yabA* results in over-initiation of DNA replication, this does not impair cell viability [7,9,11, 13].

Another regulator of DNA replication initiation is the *parABS* system. ParB is a CTP-dependent DNA sliding clamp that binds to *parS* sites to regulate ParA (Walker-type ATPase) activities with DnaA [15,23]. Deletion of any of the *parABS* system led to premature initiation of DNA replication, but this too is not detrimental to cell growth [16,23,27]. Furthermore, the viability of a *ΔyabA ΔparA* double mutant highlights redundancy in the regulatory pathways that regulate DNA replication initiation [7].

**Fig. 1.**
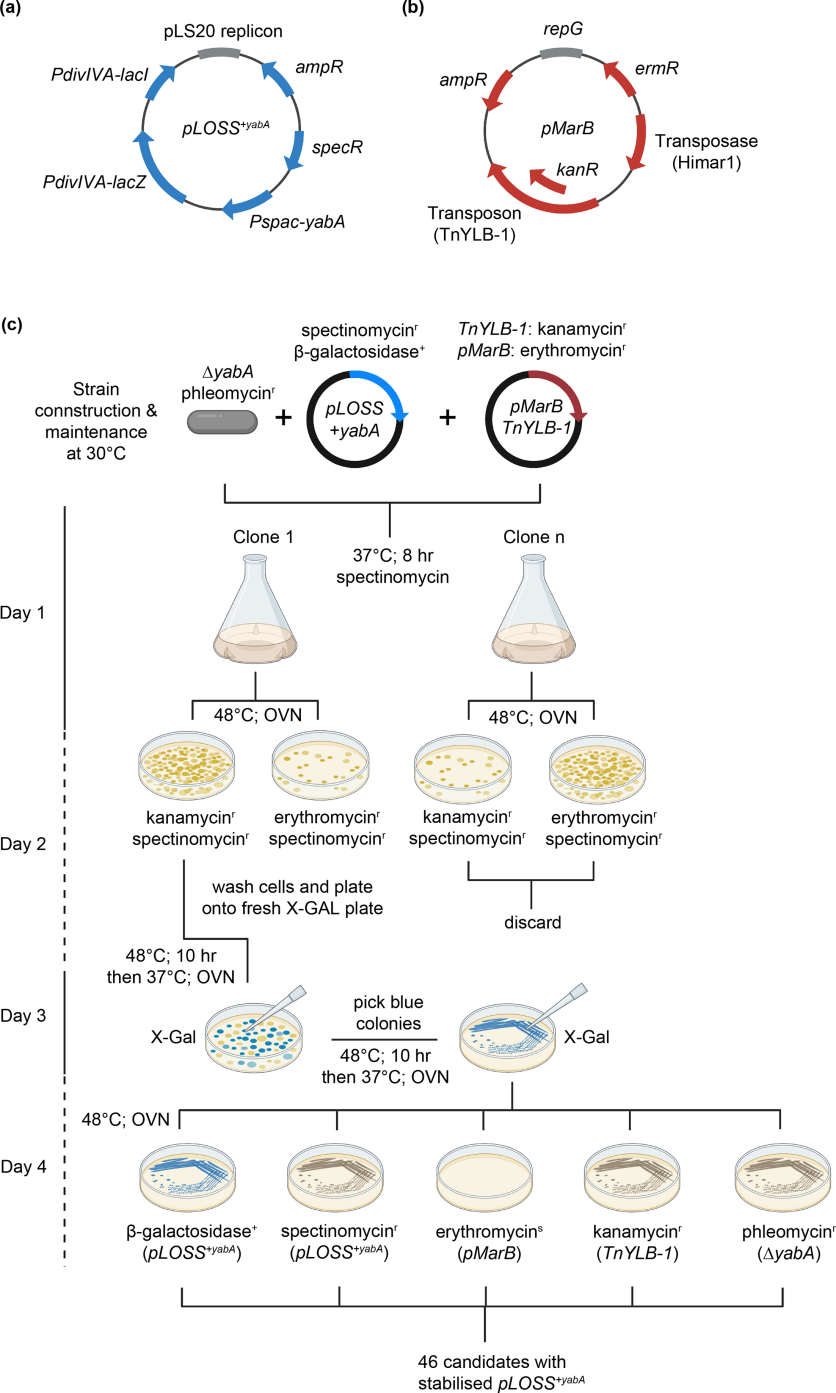
Synthetic lethal screen. (**a**) The *pLOSS* plasmid carries the *yabA* gene under the IPTG-inducible *Pspac* promoter and constitutively expresses *β*-galactosidase (*lacZ* gene) from the *PdivIVA* promoter. (**b**) The *pMarB* plasmid carries the transposase (Himar1) and transposon (TnYLB-1). (**c**) Schematic of the synthetic lethal screen. Details are provided in the ‘Experimental procedures’ section. OVN denotes overnight incubation.

**Fig. 2.**
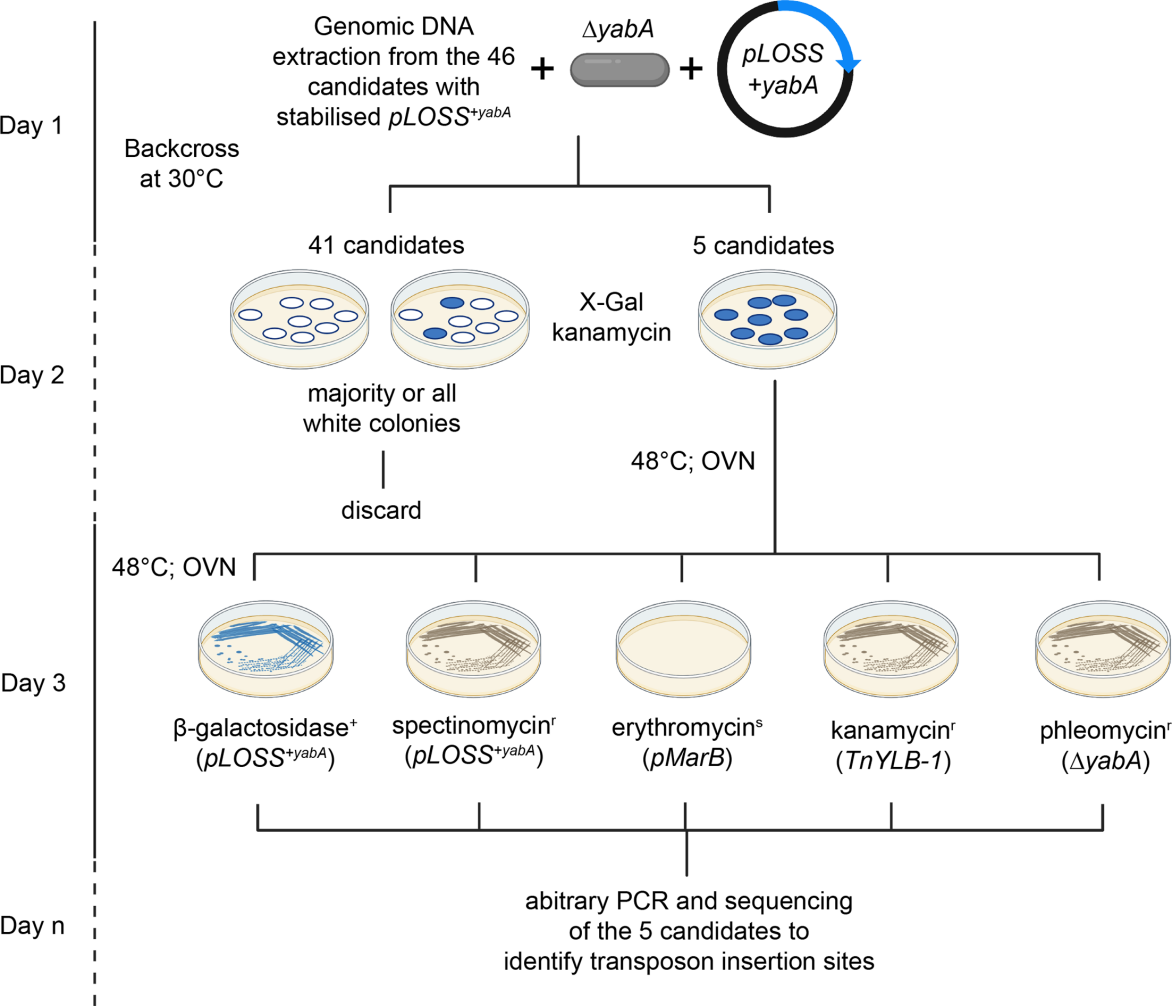
Validation of candidates from the synthetic lethal screen. Schematic of the validation steps taken after the isolation of candidates from the synthetic lethal screen. Validated candidates undergo arbitrary PCR and sequencing to identify transposon insertion sites. Details are provided in the ‘Experimental procedures’ section. OVN denotes overnight incubation.

To investigate this redundancy in regulatory mechanisms, a synthetic lethal screen exploiting the over-replicating phenotype of a *ΔyabA* mutant was used to uncover additional genes and proteins involved in the regulation of DNA replication initiation (Figs 1 and 2). In the synthetic lethal screen, the *ΔyabA* mutant was complemented by the unstable plasmid *pLOSS^+yabA^,* which carries a functional copy of *yabA*. Cells that fail to lose the plasmid after transposition suggest that the transposon insertion is not tolerated in the absence of YabA.

This manuscript describes the identification of YybE, a putative member of LTTRs, which are typically associated with metabolic regulation, as a possible factor involved in DNA replication initiation. The *ΔyabA ΔyybE* mutant retains *pLOSS^+yabA^*, suggesting that YybE confers an advantage to cells experiencing over-initiation of DNA replication in the absence of YabA. However, subsequent analyses revealed that this observation was a false positive. No significant changes in DNA replication initiation, chromosome segregation or overall chromosome morphology were detected upon deletion of YybE.

## Results

### The synthetic lethal screen

To identify candidates, a synthetic lethal screen was designed to isolate mutants that are involved in regulating the initiation of DNA replication. The over-initiation phenotype of the ∆*yabA* mutant was exploited and complemented with the unstable *pLOSS^+yabA^* (lethal or synthetic sick) plasmid, which encodes the *yabA* gene under an IPTG-inducible promoter (+) [28]. The plasmid *pLOSS* contains several features suitable for the synthetic lethal screen, a spectinomycin resistance cassette for host selection, an unstable replicon that promotes plasmid loss in the absence of selective pressure and constitutive expression of the *lacZ* gene (*β*-galactosidase) under the P*_divIVA_* promoter to enable blue/white selection on X-Gal plates (Fig. 1a) [28]. Under inducible conditions but without antibiotic selective pressure, the *ΔyabA* cells readily lose the *pLOSS^+yabA^* plasmid, consistent with YabA being a non-essential gene. To induce transposition, the plasmid *pMarB* expressing the Tn*YLB*-1 transposon was used (Fig. 1b) [29]. The plasmid *pMarB* contains several key elements for random transposition: an erythromycin resistance cassette for initial host plasmid selection, a temperature-sensitive replicon that allows curing of the plasmid after transposition, a Himar1 mariner transposase gene to mediate transposition of the TnYLB-1 transposon into random TA dinucleotide sites and a TnYLB-1 transposon that carries the kanamycin resistance cassette to allow selection after chromosomal insertions (Fig. 1b) [29].

Generation of the library was performed using the *ΔyabA* mutant harbouring both plasmids (*pLOSS^+yabA^ and pMarB*) and yielded a library containing 70,000+transposon-inserted mutants (see the ‘Experimental procedures’ section for detail) (Fig. 1c). A series of validation tests was performed to eliminate false positives (see the ‘Experimental procedures’ section for a detailed explanation), which yielded 46 mutant candidates that formed blue colonies and were erythromycin-sensitive, but spectinomycin-, kanamycin- and phleomycin-resistant (Fig. 1c). These candidates appeared to retain *pLOSS^+yabA^* without antibiotic selective pressure. Next, these 46 mutations were backcrossed into the parent ∆*yabA pLOSS^+yabA^* to confirm that *pLOSS^+yabA^* retention was due to transposon insertion and not due to secondary mutation within the genome or the integration of *pLOSS^+yabA^* into the genome (Fig. 2). Of the 46 candidates after backcrossing, 5 candidates consistently produced blue colonies and are erythromycin-sensitive, but spectinomycin-, kanamycin- and phleomycin-resistant (Fig. 2).

### Identification of YybE as a candidate

**Fig. 3.**
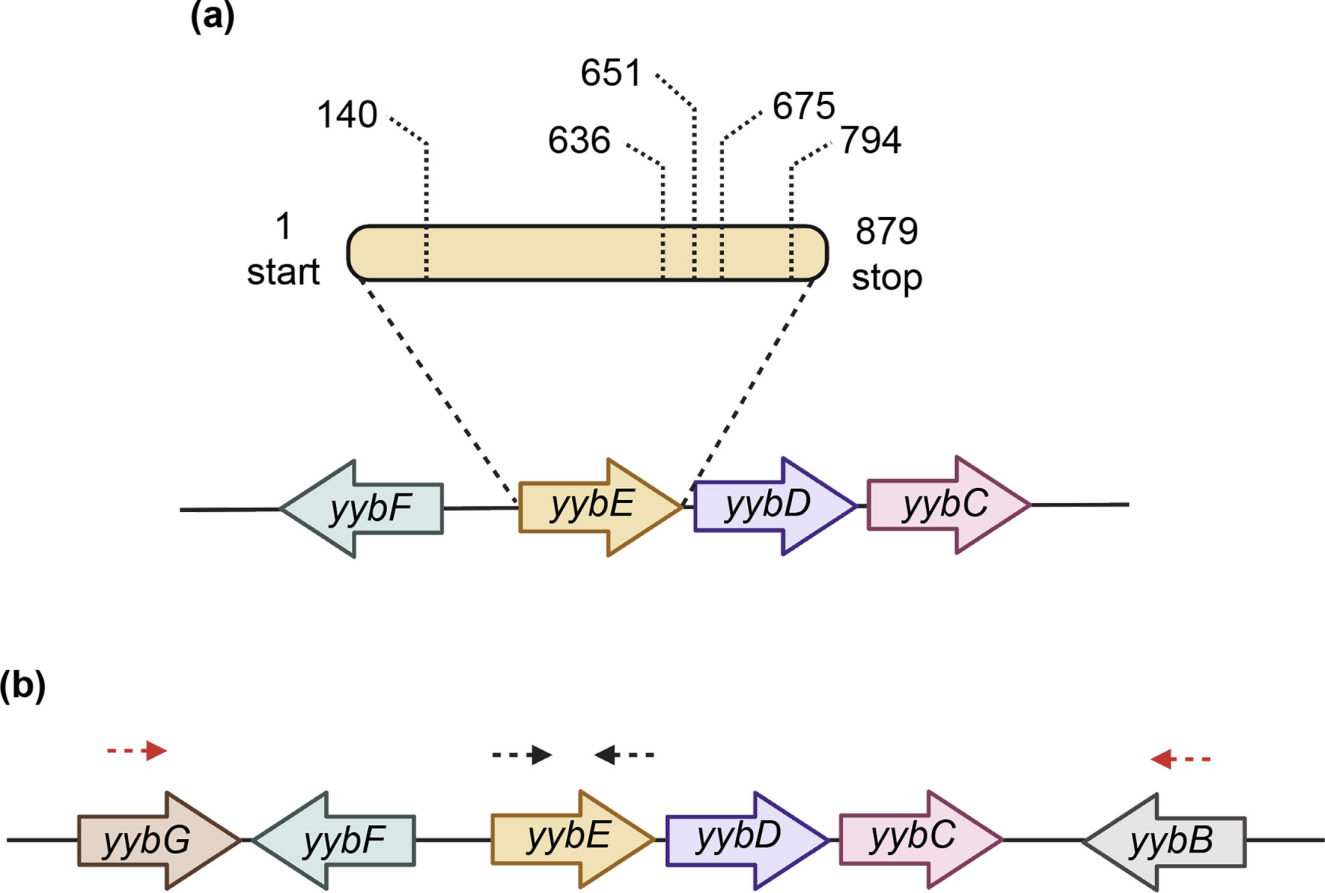
Identification of YybE as a candidate. (**a**) Transposon insertions were mapped within the uncharacterized gene *yybE* at positions 140, 636, 651, 675 and 794. Schematic of the genomic neighbourhood of *yybE. yybE* forms an operon with *yybD* and *yybC* and lies upstream of *yybF* on the opposite strand. (**b**) Location of primer pairs designed to confirm transposon insertions within *yybE*. oAK3A/oAK4A amplifies the entire *yybE* ORF (black arrow), while oAK3B/oAK4B amplifies the region, including *yybF*, *yybE*, *yybD* and *yybC* (red arrow).

Arbitrary PCR and DNA sequencing were performed to identify the transposon insertion sites (Fig. 2). All five transposon insertions map to an uncharacterized gene, *yybE,* at amino acid position 140, 636, 651, 675 and 794, respectively (Fig. 3a). Based on information from Subtiwiki, *yybE* likely forms an operon with the downstream genes *yybD* and *yybC*, while *yybF* is located on the opposite strand upstream of *yybE* (Fig. 3a). To confirm that the transposon insertions were within *yybE*, primers were designed to specifically amplify the entire *yybE* ORF (Fig. 3b). PCR confirmed that all candidates carried insertion within *yybE* and that no intact copy of *yybE* was duplicated elsewhere in the genome. To rule out the possibility that transposon insertion at *yybE* might have disrupted the neighbouring genes and contributed to the stabilization of the *pLOSS^+yabA^* plasmid, the region spanning the ORFs of *yybF*, *yybE*, *yybD* and *yybC* was also amplified, followed by DNA sequencing. Analysis confirmed that transposon insertions were confined to the *yybE* ORF and that the ORFs and the intergenic regions between *yybF*, *yybD* and *yybC* remained intact (Fig. 3b). While polar effects on neighbouring genes caused by the insertion cannot be entirely excluded, they are less likely in *Bacillus subtilis* as transcription and translation are not tightly coupled and rely mainly on Rho-independent intrinsic terminators that act at defined positions near the ends of genes [30]. As a result, disrupting translation upstream does not usually trigger premature termination, which allows downstream genes to remain expressed [30].

### YybE possesses functional domains characteristic of the LTTRs

**Fig. 4.**
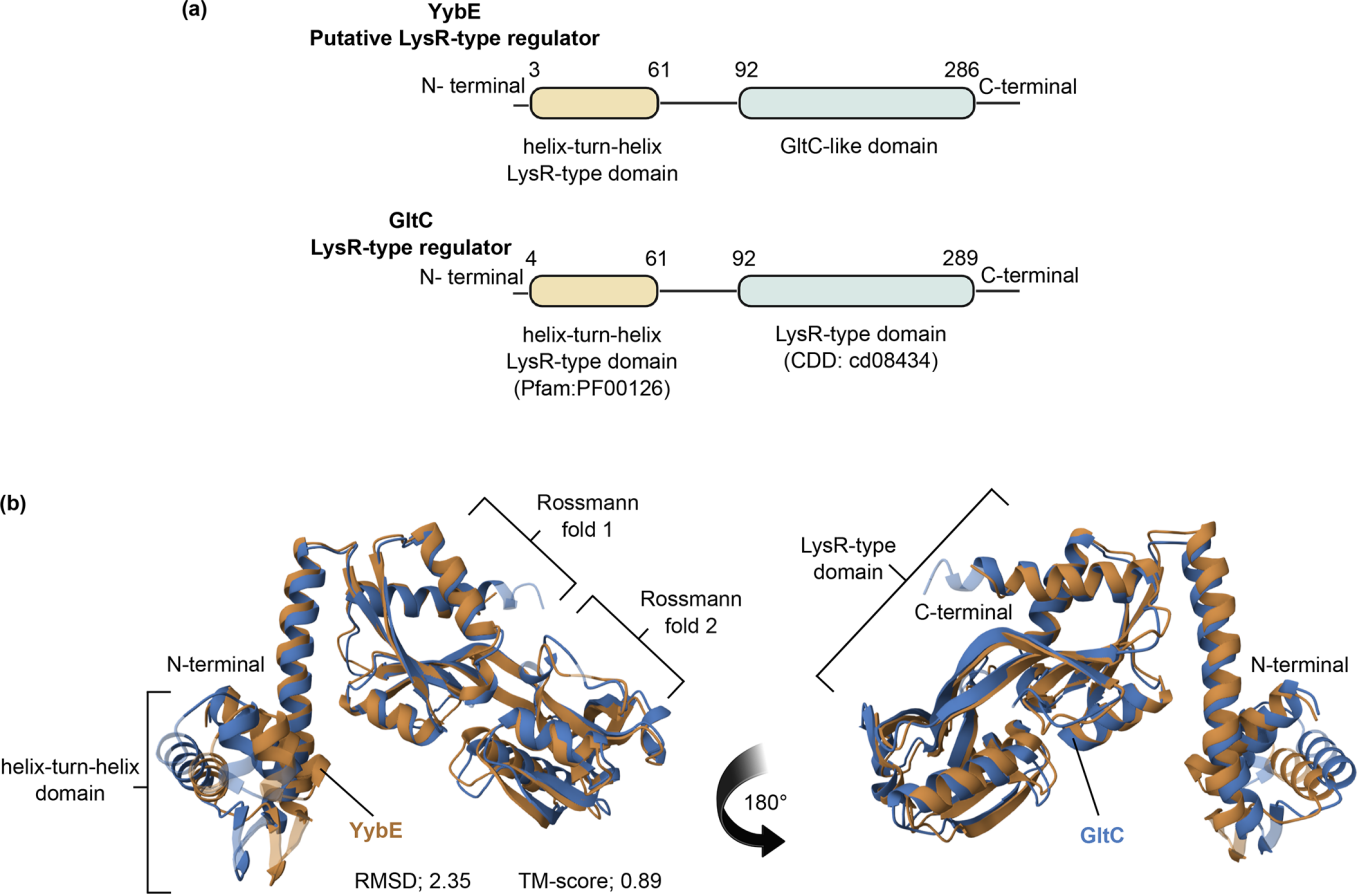
Functional domain prediction and structural alignment of YybE. (**a**) Functional domain analysis predicts that YybE contains a helix-turn-helix domain (Pfam: PF00126) from residues 3 to 61 and a GltC-like domain (CDD: cd08434) from residues 92 to 286. Identification of functional domains was performed by InterPro. (**b**) The structure of YybE (orange) was superimposed onto GltC (blue) revealing a high degree of similarity between the two proteins (RMSD, 2.35; and TM-score, 0.89). Structural alignment was analysed by the RCSB PDB Pairwise Structure Alignment tool using protein homology models generated by AlphaFold (YybE, AF-P37499-F1-v4; and GltC, AF-P20668-F1-v4).

To investigate further into YybE, its sequence was analysed for functional domains with the InterproScan tool in combination with the Pfam and CDD databases. The analysis identified two key domains: the first is a helix-turn-helix domain located at residues 3 to 61, which corresponds to the bacterial regulatory helix-turn-helix (HTH) protein family (Fig. 4a). The second is a GltC-like effector binding domain spanning residues 92 to 286 (Fig. 4a). Interestingly, GltC belongs to the LysR-type family of transcriptional regulators that regulate the expression of the *gltAB* operon involved in glutamate synthesis [3,5, 31]. These two key domains identified in YybE are characteristics of LTTRs [1,2].

To validate these functional domains, a structural comparison was performed between the AlphaFold predicted structures of YybE and GltC (Fig. 4b). The structural models were overlaid using the RCSB PDB pairwise structure alignment tool, which revealed significant structural conservation between the HTH domain and the effector binding domain. Further structural analysis of YybE revealed that the effector-binding domain contains two conserved Rossmann folds that are characteristic of LTTRs, which further supported the notion that YybE is a putative LTTR (Fig. 4b) [1,2, 32].

### Evolutionary and structural conservation place YybE within the LTTR family

**Fig. 5.**
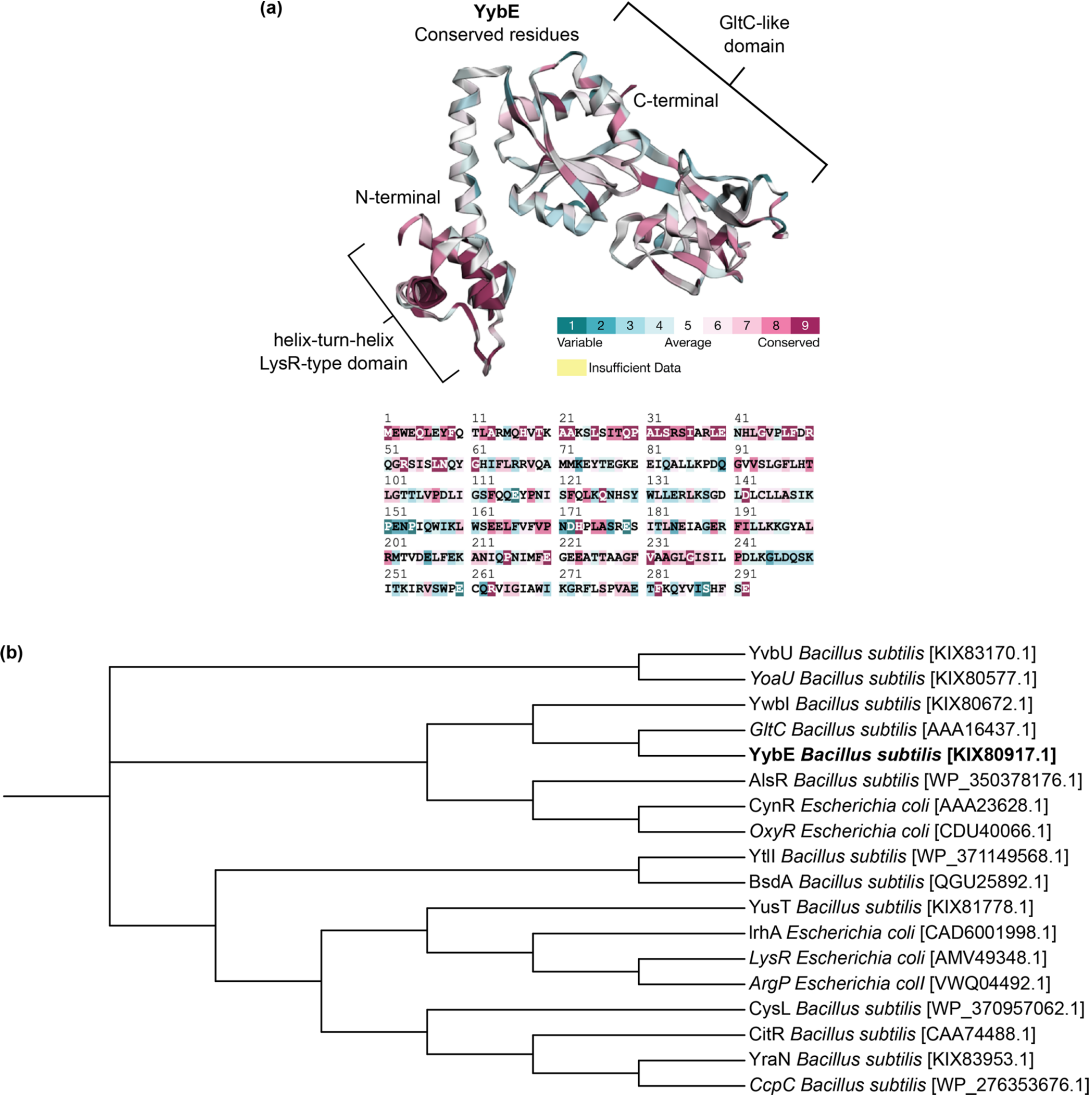
Conservation and phylogenetic analysis of YybE. (**a**) The conservation of amino acid residues in YybE was mapped onto its protein structure with conservation scores ranging from 1 (variable, cyan) to 9 (highly conserved, dark red). Highly conserved residues are located across the DNA-binding domain (HTH-domain) and regulatory domain (LysR-type domain). Evolutionary conservation was analysed by Consurf and mapped onto the structure generated by AlphaFold (YybE: AF-P37499-F1-v4). (**b**) Phylogenetic analysis of YybE (highlighted in bold) clusters it within the LysR-type family of transcriptional regulators. A phylogenetic tree was visualized with iTOL using sequence alignment performed by MAFFT.

The evolutionary conservation of the domains was next explored using the ConSurf server to map evolutionary conservation onto the YybE protein structure based on multiple sequence alignment of LTTR proteins. Both the HTH domain and the effector binding domain showed high conservation (Fig. 5a). Additionally, to establish the evolutionary relationship of YybE within the LTTR family, a phylogenetic tree was constructed by aligning the protein sequence of YybE with known/putative LTTR family members using MAFFT. The tree revealed that YybE clustered with GltC, suggesting functional and evolutionary relevance with LTTRs (Fig. 5b).

LTTRs are known for their role in regulating diverse metabolic processes; therefore, YybE might influence DNA replication initiation through the regulation of metabolic genes. Bacterial metabolic states, in turn, modulate DNA replication-associated proteins such as YabA and ParA. Therefore, it was plausible that YybE might play a role in regulating DNA replication initiation via metabolic states.

### YybE does not influence the initiation of DNA replication

**Fig. 6.**
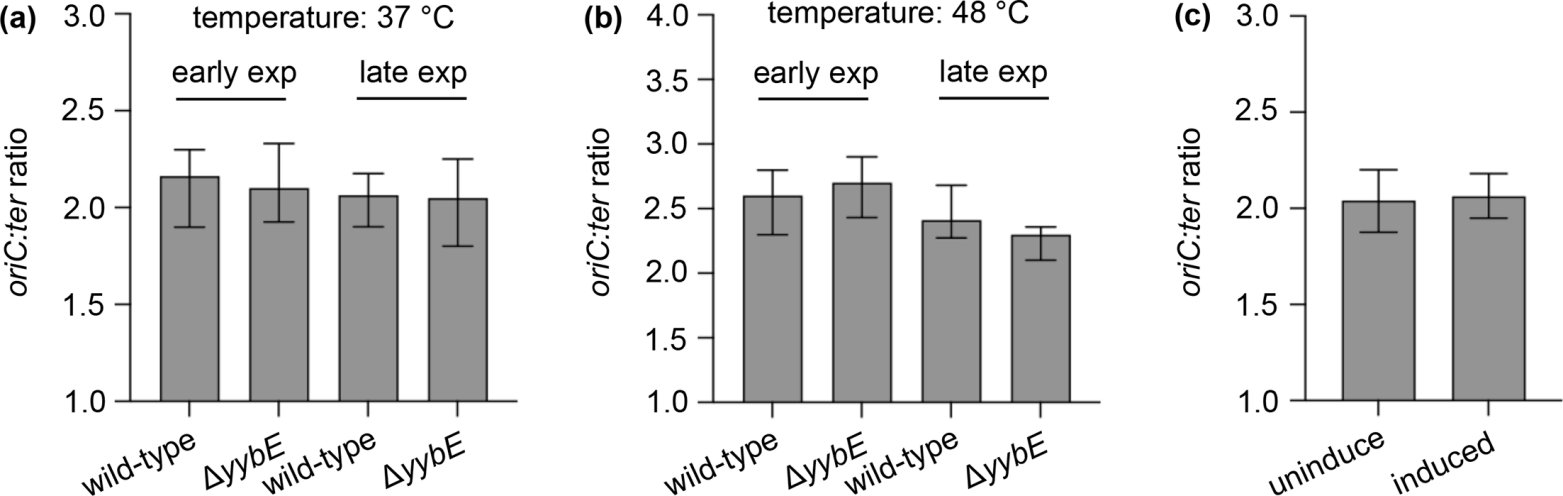
Normal frequency of DNA replication initiation in the *yybE* mutant. The frequency of DNA replication initiation was not affected (**a**) under the different growth phases (early and late exponential phase), (**b**) temperatures (37 °C and 48 °C) and (**c**) overexpression of *yybE*. Overexpression was achieved by induction with 1% xylose. The *oriC:ter* ratio was determined by quantitative PCR.

To investigate the possible role of YybE in DNA replication initiation, and to rule out the possibility of the *pLOSS^+yabA^* plasmid affecting analysis, the ORF of *yybE* was deleted in the wild-type *B. subtilis* strain 168 by replacing it with a kanamycin resistance marker (see the ‘ Experimental procedures’ section). The initiation rates of DNA replication across different growth phases were measured with marker frequency analysis. The *ori:ter* ratio showed no significant change in the absence of YybE (Fig. 6a).

During the construction of the library, 48 °C was used for transposition. It was, therefore, possible that when the elevated temperature combined with the inherent instability of the AT-rich DUE (DNA unwinding element), the origin favoured an open complex formation [33]. Furthermore, YybE processes the DNA-binding domain; hence, YybE might bind to the AT-rich region of the DUE and promote DUE stability. To test this possibility, mutants were grown at 48 °C, and the initiation rate of DNA replication was measured. Again, no significant alteration in the *ori:ter* ratio was detected (Fig. 6b). Additionally, overexpression of *yybE* also failed to alter the *ori:ter* ratio (Fig. 6c).

### Chromosome morphology remains unaffected by YybE

**Fig. 7.**
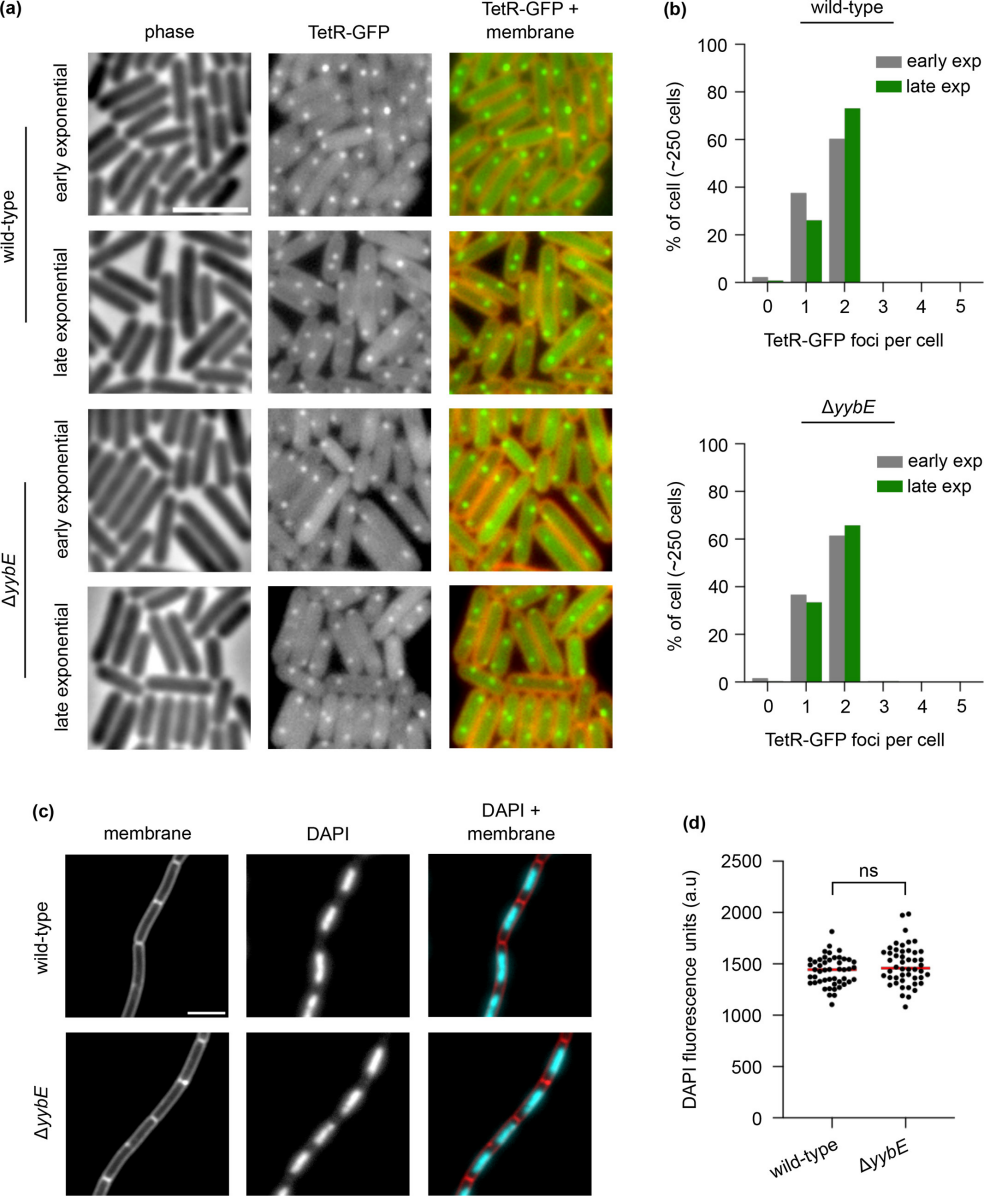
Normal chromosome origin segregation and chromosome organization in the *YybE* mutant. (**a**) Origin localization in a wild-type strain and a *yybE* mutant during the early and late exponential growth phases. The origin region was labelled with a *tetO* array (~150 operator sites) bound by TetR-GFP. Phase contrast (left panel), TetR-GFP (middle panel) and membrane dye FM 5-95 overlayed with TetR-GFP (right panel). (**b**) The majority of cells have two origins per cell, regardless of growth phase. (**c**) DAPI staining of chromosomal DNA. Membrane dye FM 5-95 (left panel), DAPI stain (middle panel) and membrane dye overlayed with DAPI staining (right panel). (**d**) DAPI fluorescence intensity remains unchanged in the *yybE* mutant, *n*=50 cells. Median intensities are indicated with red lines, together with *P*-values of an unpaired, two-sided t-test; ns, not significant. Scale bar, 3 µm.

It was plausible that deletion of *yybE* might cause a small subpopulation of cells to undergo asynchronous DNA replication; hence, marker frequency analysis was not sensitive enough to detect the changes. To investigate this in greater detail, strains were constructed in which the chromosomal origin region was visualized using a fluorescent repressor operator system. This system employs TetR-GFP bound to a *tetO* array (~150 *tetO* operator sites) located at the *oriC*, enabling single-cell analysis to directly observe and quantify DNA replication initiation events. In agreement with the marker frequency analysis, the majority of cells exhibited two TetR-GFP foci per cell (Fig. 7a and b). Furthermore, as with typical wild-type *B. subtilis oriC* localization, the ∆*yybE* mutant also displayed two TetR-GFP foci near the cell poles, suggesting normal origin segregation (Fig. 7a) [24]. Next, DAPI staining was used to visualize the nucleoid, and as with wild-type cells, *yybE* mutants exhibit normal chromosome morphology, and DNA content remains unchanged (Fig. 7c and d). These observations suggest that YybE does not affect DNA replication initiation and chromosome morphology under the experimental conditions tested.

### The YabA/YybE double mutant did not further increase DNA replication

**Fig. 8.**
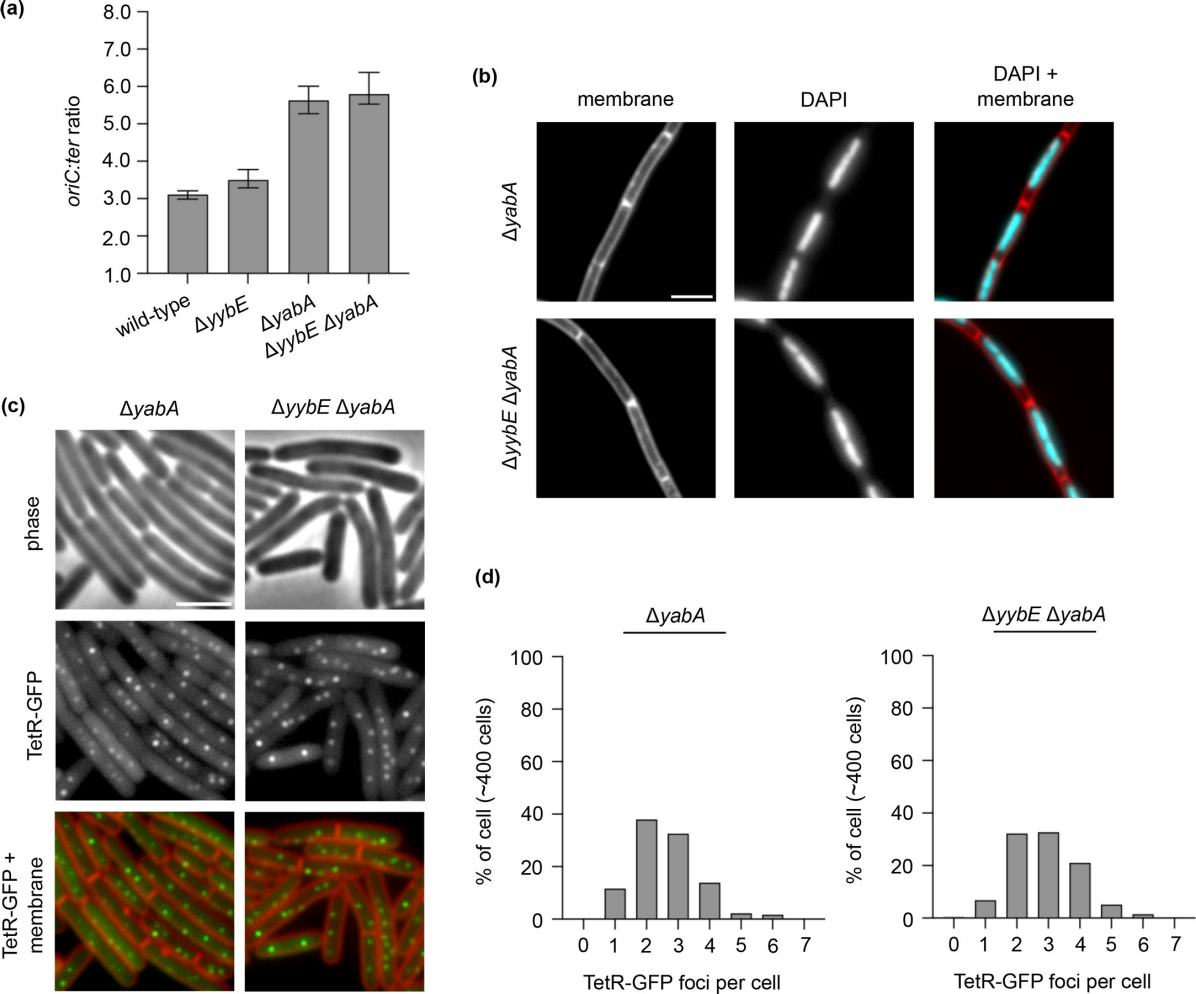
DNA replication initiation frequency, chromosome organization and chromosome origin segregation were not disrupted in the *yybE yabA* mutant. (**a**) The frequency of DNA replication initiation was not affected. The *oriC:ter* ratio was determined by quantitative PCR. (**b**) DAPI staining of chromosomal DNA indicates that chromosome organization was not affected. Membrane dye FM 5-95 (left panel), DAPI stain (middle panel) and membrane dye overlayed with DAPI staining (right panel). (**c**) The origin localization of the mutants. Origin region was labelled with an array of tetO operators bound by TetR-GFP. Phase contrast (top panel), TetR-GFP (middle panel) and membrane dye FM 5-95 overlayed with TetR-GFP (bottom panel). (**d**) YabA dependent increase in the number of origins per cell. Scale bar, 3 µm.

During library construction, confirmed candidate mutants with transposon insertion all retained the *pLOSS^+yabA^* plasmid without antibiotic selective pressure. This suggests that retaining YabA in the absence of YybE is beneficial to the cell. Therefore, the ∆*yabA* ∆*yybE* double mutant might be synthetically sick. Surprisingly, the ∆*yabA* ∆*yybE* double mutant was viable and did not exhibit a more pronounced defect in DNA replication initiation and chromosome morphology compared to the ∆*yabA* single mutant under the conditions tested (Fig. 8). The increase in the *ori:ter* ratio and TetR-GFP foci is consistent with YabA being a negative regulator of DNA replication initiation [8,9].

### The isolated transposon insertion mutants were false positives

**Fig. 9.**
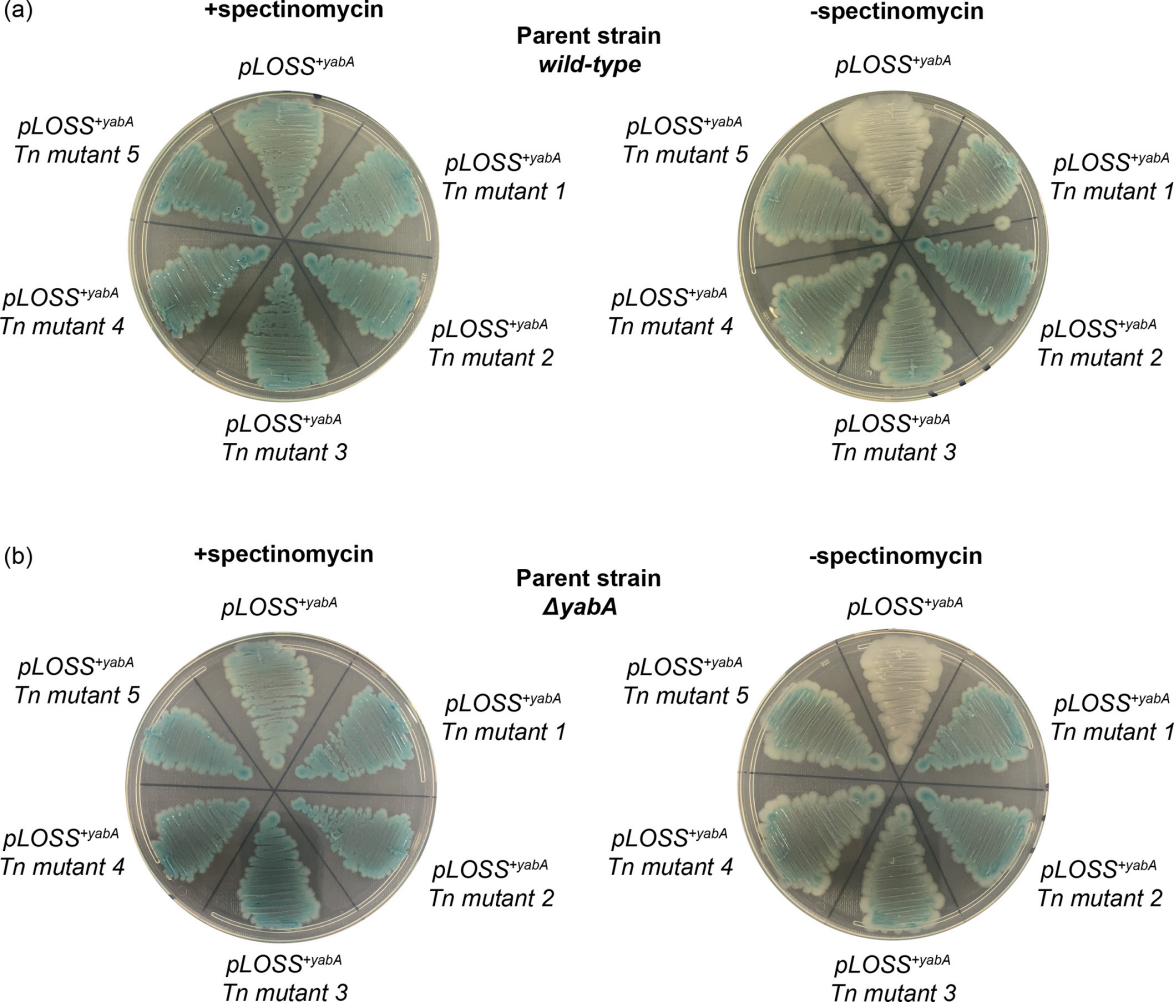
The isolated transposon insertion mutants were false positives. The *pLOSS^+yabA^* plasmid was extracted from the five transposon insertion candidates and transformed into (**a**) the wild-type *B. subtilis* strain 168 and (**b**) the *ΔyabA* mutant. The resulting transformants were streaked over multiple generations on X-GAL agar plates in the presence or absence of spectinomycin. Transformants that retained the *pLOSS^+yabA^* plasmid formed blue colonies.

The viability of the *∆yabA ∆yybE* double mutant suggests that the five transposon insertion mutants isolated during the screen were false positives. To verify this, the *pLOSS^+yabA^* plasmids were recovered from each of the five transposon insertion mutants and subsequently transformed into both the wild-type *B. subtilis* strain 168 and the parental *∆yabA* mutant. The recovery of intact plasmids from all five transposon insertion mutants strongly suggests that plasmid integration into the chromosome is unlikely to account for the false-positive phenotype. Plasmid stability was then assessed by streaking the transformants over multiple generations in the absence of antibiotic selection. Consistent with the false-positive interpretation, transformants harbouring the *pLOSS^+yabA^* plasmid isolated from the transposon mutants remained blue without antibiotic selective pressure, whereas the parental *pLOSS^+yabA^* plasmid was readily lost under the same condition (Fig. 9). These findings suggest that retention of the *pLOSS^+yabA^* plasmid is likely caused by mutation(s) on the plasmid which affect plasmid stability, copy number and/or segregation efficiency, independent of *yybE*.

## Discussion

Deletion of known regulators of DNA replication, such as YabA and ParA, results in over-initiation of DNA replication, yet the viability of the *ΔyabA ΔparA* double mutant raises the possibility of additional regulatory mechanisms regulating DNA replication initiation [7]. In this study, a transposon mutagenesis screen has identified *yybE*, encoding a putative LysR-type transcriptional regulator, as a potential candidate. However, no detectable changes in DNA replication initiation or chromosome morphology were observed in the absence of YybE, and subsequent verification confirmed that the five transposon insertion candidates from the screen were false positives. The retention of the covering plasmid independent of *yybE* is most likely due to a stabilizing mutation(s) within the plasmid, which potentially increases its copy number, stability and/or segregation efficiency. Any of these effects would promote plasmid retention in the absence of selective pressure.

Future studies utilizing covering plasmids should implement steps to minimize false positives, such as recovering plasmids or transposon insertion alleles from candidates and reintroducing them into the parental background to distinguish true genetic interactions from phenotypes caused by plasmid-stabilizing mutations or plasmid integration into the genome. Furthermore, analysis of transposon insertion sites to determine whether the disrupted gene lies within an operon would enable assessment of potential polar effects caused by neighbouring genes, which could be tested experimentally through operon deletion or complementation approaches.

## Experimental procedures

### Strains and growth conditions

All strains used in this study are listed in Table S1 (available in the online Supplementary Material) and were maintained on nutrient agar (Oxoid) with supplements and antibiotics. Selective media contained 2 µg ml^−1^ kanamycin, 50 µg ml^−1^ spectinomycin, 1 µg ml^−1^ erythromycin, 1 µg ml^−1^ phleomycin, 100 µg ml^−1^ ampicillin, 40 µg ml^−1^ X-Gal and 1 mM IPTG. For experiments in *B. subtilis*, cells were grown in casein hydrolysate medium (CH) (Merck).

### DNA manipulation and strain constructions

*B. subtilis* transformations were carried out as previously described [34,35]. DNA for transformation was provided as either *B. subtilis* genomic DNA or a linearized plasmid containing the desired genetic construct. Plasmid extractions were carried out according to the manufacturer’s protocol (Qiagen Miniprep Kit or NEB Monarch Plasmid Miniprep Kit). Information on strains and plasmids is listed in Tables S1 and S2, respectively.

### Generation of plasmids *pLOSS^+yabA^* and *pSG1728E*

The plasmids were constructed by amplifying the ORF of the *yabA* (primer oAK1/oAK2) and *yybE* (primer oAK5/oAK6) gene with Phusion high-fidelity DNA polymerase (Thermo Fisher Scientific) from 168ED gDNA. This would introduce the following restriction sites: BmtI and SphI (*yabA* insert) and AvrII and XhoI (*yybE* insert) at the 5′ and 3′ ends, respectively. Restriction digestion of the amplified inserts and vector plasmids was performed. The digested inserts were purified using the PCR purification kit (QIAGEN) to stop the digestion reaction, and the vector was treated with shrimp alkaline phosphatase (NEB) to prevent re-ligation of the linearized vector. T4 DNA ligase (NEB) was used to catalyse the joining of the insert with the vector to generate inducible *yabA* expression under the *Pspac* promoter (*pLOSS^+yabA^*) and inducible *yybE* expression under the *Pxyl* promoter (*pSG1728E*).

### Generation of the *yybE* deletion strain

To construct the *yybE* deletion strain, overlap extension PCR was performed with Phusion high-fidelity DNA polymerase (Thermo Fisher Scientific), consisting of three fragments of DNA with ~20 bp of overlaps. The left kanamycin fragment is 230 bp (oAK7/oAK8), consisting of 44 bp upstream of the *yybF* stop codon and the intergenic region until before the start codon of *yybE* from 168ED gDNA. The right kanamycin fragment is 224 bp (oAK11/oAK12), consisting of the intergenic region immediately after the stop codon of *yybE* until 211 bp into *yybD* from 168ED gDNA. The kanamycin cassette fragment (oAK9/oAK10) of 1,442 bp was amplified from pHM23. The three DNA fragments were purified using the PCR purification kit (QIAGEN) and stitched together as in a normal PCR reaction, but without primers. The resulting DNA fragment was then used as a template for PCR with primers (oAK7/oAK10). The amplified fragment was then PCR purified (QIAGEN) and transformed into 168ED, yielding AK36 (*DyybE::neo*). Deletion of *yybE* was confirmed by PCR (oAK3A/oAK4A) and resistance to kanamycin.

### Synthetic lethal screen

To identify mutants synthetically lethal in the absence of *yabA*, a two-step transformation was performed at 30 °C. The plasmid *pLOSS^+yabA^* was first introduced into strain AK40 (*yabA::phl*) to generate HM687 (*yabA::phl pLOSS^+yabA^*). This strain was then transformed with the transposon plasmid *pMarB* while maintaining selection for *pLOSS^+yabA^* (spectinomycin), yielding strain AK1E (*ΔyabA::phl pLOSS^+yabA^ pMarB*). Independent transformants were grown individually at 37 °C in LB supplemented with spectinomycin and IPTG for 8 h and then plated onto selective agar plates and grown at 48 °C overnight containing either spectinomycin and kanamycin or spectinomycin and erythromycin (Fig. 1). The next day, to select for successful transposition events, plates showing the highest ratio of kanamycin resistance to erythromycin resistance colonies and colony size variation were selected to generate the transposon library. Colonies from the kanamycin and spectinomycin plates were collected and washed with LB and replated on X-Gal and IPTG plates. These plates were then incubated for 10 h at 48 °C and then transferred to 37 °C overnight. The next day, blue colonies were picked and streaked on fresh X-Gal and IPTG plates for a second round of incubation at 48 °C for 10 h and then overnight at 37 °C to eliminate false positives. Candidates that remained blue were tested on plates containing IPTG and various antibiotics (kanamycin; *TnYLB*-1, erythromycin; *pMarB*, spectinomycin; *pLOSS^+yabA^*, phleomycin; *∆yabA* and X-Gal; *pLOSS^+yabA^*) (Fig. 1). Candidates that were blue, erythromycin-sensitive, spectinomycin-, kanamycin- and phleomycin-resistant were backcrossed into the parent strain, HM687 (Fig. 2). Individual transformants then undergo a further round of antibiotic-resistant tests, and the resulting candidates were subjected to a two-step arbitrary PCR reaction to amplify the transposon insertion region (primer: MarB1N/Arb1A), followed by a second round of more precise PCR (primer: MarB1/Arb2A). The amplified regions were sequenced to map the transposon insertion sites.

### Bioinformatic analysis

Functional domain identification: the protein sequence of YybE (Subtiwiki; BSU_40670) was analysed by the InterProScan tool to predict functional domains [36,38]. The Pfam and CDD databases were then used to identify functional domains [39,40]. Protein structural analysis: the predicted structures of YybE (AF-P37499-F1-v4) and GltC (AF-P20668-F1-v4) were retrieved from the AlphaFold protein structure database [41,42]. The protein structures were aligned with the RCSB PDB pairwise structure alignment tool [43]. Protein conservation analysis: the evolutionary conservation of YybE was assessed by retrieving homologous sequences from the UniProt database [44]. The level of conservation was mapped onto the AlphaFold predicted model of YybE by Consurf to highlight regions of conservation [45,47]. Phylogenetic tree: the evolutionary relationship of YybE within the LTTR family was analysed by multiple sequence alignment of YybE with known and putative LTTR proteins using MAFFT-DASH (YybE; KIX80917.1, CysL; WP_370957062.1, GltC; AAA16437.1, AlsR; WP_350378176.1, BsdA; QGU25892.1, CcpC; WP_276353676.1, CitR; CAA74488.1, YoaU; KIX80577.1, YraN; KIX83953.1, YtlI; WP_371149568.1, YusT; KIX81778.1, YvbU; KIX83170.1, YwbI; KIX80672.1, ArgP; VWQ04492.1, CynR; AAA23628.1, lrhA; CAD6001998.1, LysR; AMV49348.1, OxyR; CDU40066.1) [48]. The interactive Tree of Life (iTOL) was then used to visualize the phylogenetic tree [49].

### Marker frequency analysis

Measurement of DNA replication was done with cultures grown overnight at 37 ˚C in CH medium (Merck) without antibiotics, before resuspending in fresh CH medium the following day. The cultures were grown to either early or late exponential phase at 37 ˚C and 0.5% of sodium azide (Sigma) was added to prevent further metabolism. Chromosomal DNA was isolated with a DNeasy Blood and Tissue Kit (QIAGEN). Quantitative PCR was performed with Rotor-Gene SYBR Green (QIAGEN) in a Rotor-Gene Q Instrument (QIAGEN). For origin quantification, the intergenic region between *dnaA* and *dnaN* was amplified using primers oqPCR1 and oqPCR2 (Table S3). For terminus quantification, the region downstream of *yocG* was amplified using primers oqPCR3 and oqPCR4 (Table S3). The relative quantification analysis (ΔΔCT) was calculated with Rotor-Gene Software version 2.0.2 (QIAGEN) to determine the *ori:ter* ratio of each test sample. Samples were normalized to the *ori:ter* ratio of *B. subtilis* spore DNA. At least two biological repeats were performed, with error bars indicating the standard deviation of at least three technical replicates from one experimental set.

### Epifluorescence microscopy

Fluorescent microscopy analyses were done with cells grown at 37 °C in accordance with the marker frequency analysis. Cell membranes were stained with 0.4 µg ml^−1^ FM 5–95 membrane dye from Thermo Fisher Scientific. Chromosomes were stained with 10 µg ml^−1^ DAPI from Thermo Fisher Scientific [50]. Cells were immobilized onto a 1.2% agarose pad in 0.5 × CH medium. Microscopy was carried out with the Nikon Eclipse Ti equipped with a Nikon DM 100×/1.40 oil Ph3 objective, a Photometrics CoolSnap HQ2 CCD camera and a Lambda LS light source (Sutter Instrument). Image acquisitions were carried out with Metamorph V.7.7. Cells were quantified using Fiji [51]. At least three biological repeats were performed for each experiment.

### Fluorescent intensity measurement

DAPI fluorescent intensity was measured by performing background subtractions on the images, and the fluorescent intensity was generated by aligning perpendicular to the cell length axis and by measuring along the length axis of the cell. Cells were quantified using Fiji [51].

## Supplementary material

10.1099/acmi.0.001000.v4 Uncited Table S1.

## Abbreviations

HTH: helix-turn-helix
LTTR: LysR-type transcriptional regulator
