# Supplementary material for: The Bacillus subtilis putative LysR-type transcriptional regulator YybE and its connection to chromosome replication and segregation
Source: Access Microbiol. 2026 Jan 7;8(1):001000.v4. doi: 10.1099/acmi.0.001000.v4 (PMC12779278; doi:10.1099/acmi.0.001000.v4)
Supplement: Uncited Table S1. [file acmi-8-01000-s001.pdf]

Supplementary Table 1: Strains used in this study

Strains	Genotype[a]	Construction[b]	Source
B. subtilis 168ED	*trpC2 wild type*		[1]
B. subtilis 168CA	*trpC2 wild type*		[2]
B. subtilis AK1E	*trpC2 ΔyabA::phl pLOSS^{+yabA} pMarB*	pMarB → HM687	this work
B. subtilis AK32	*trpC2 amyE::Pxyl-yybE(spc)*	pSG1728E → 168ED	this work
B. subtilis AK36	*trpC2 ΔyybE::neo*		this work
B. subtilis AK40	*trpC2 ΔyabA::phl*	NR19 → 168ED	this work
B. subtilis AK42	*trpC2 ΔyabA::phl ΔyybE::neo*	AK36 → AK40	this work
B. subtilis AK47	*trpC2 tetR-gfp(spc)/tetO^{-7}(ery)*		[3]
B. subtilis AK49	*trpC2 tetR-gfp(spc)/tetO^{-7}(ery) ΔyybE::neo*	AK36 → AK47	this work
B. subtilis AK51	*trpC2 tetR-gfp(spc)/tetO^{-7}(ery) ΔyabA::phl*	AK40 → AK47	this work
B. subtilis AK53	*trpC2 tetR-gfp(spc)/tetO^{-7}(ery) ΔyybE::neo ΔyabA::phl*	AK36 → AK51	this work
B. subtilis HM687	*trpC2 ΔyabA::phl pLOSS^{+yabA}*	pHM399 → AK40	this work
B. subtilis NR19	*trpC2 ΔyabA::phl*		[4]

[a] *B. subtilis* antibiotic resistance markers are listed respectively. neo, kanamycin resistance; ery, erythromycin resistance; spc, spectinomycin resistance; phl; phleomycin resistance.

[b] Plasmids or genomic DNA of the indicated strain transformed into the parent strain used in the genetic construction.

Supplementary Table 2: Plasmids used in this study

Plasmids	Genotype[a]	Construction[b]	Source
pHM399/pLOSS^{+yabA}	*bla spc Pspac-yabA*	gift from Heath Murray	this work
pLOSS	see reference		[5]
pMarB	see reference		[6]
pSG1728	see reference		[7]
pSG1728E	*bla spc amyE::Pxyl-yybE*	168ED (oAK5/oAK6) → pSG1728	this work

[a] *E. coli and B. subtilis* antibiotic resistance markers are listed first and second respectively. bla, ampicillin resistance; neo, kanamycin resistance; ery, erythromycin resistance; spc, spectinomycin resistance; phl, phleomycin resistance.

[b] DNA fragments amplified with primer pair indicated in parentheses using template from genomic DNA before the amplified products are cloned into the parent plasmid used in the genetic construction.

Supplementary Table 3: Oligonucleotides used in this study

Primer	Sequence (5' → 3')
ARB1A	CCACGCGTCGACTAGTACNNNNNNNNNNGATAT
ARB2A	CCACGCGTCGACTAGTAC
MarB1N	GCGCCTACGAGGAATTTGTATC
MarB1	GTTAGACCGGGGACTTATC
oAK5	AATTTAATTTCCTAGGTTGGAATGGGAACAACTTG
oAK6	AATTTAATTTCTCGAGCTACTCAGAAAAATGGCTGATCAC
oqPCR1	GTAGGGCCTGTGGATTTGTG
oqPCR2	GATCAATCGGGGAAAGTGTG
oqPCR3	ATTCTGCTGATGTGCAATGG
oqPCR4	TCCATATCCTCGCTCCTACG

Supplementary Table 4: Strains used in each figure

Fig.1	AK1E *(ΔyabA pLOSS^{+yabA} pMarB)*
Fig.4	168ED *(wild-type)*, AK36 *(ΔyybE)*, AK32 *(Pxyl-yybE)*
Fig.5	AK47 *(tetR-gfp/tetO^{-7})*, AK49 *(tetR-gfp/tetO^{-7} ΔyybE)*
Fig.6	168ED *(wild-type)*, AK36 *(ΔyybE)*, AK40 *(ΔyabA), AK42 (ΔyabA ΔyybE),* AK51 *(tetR-gfp/tetO^{-7} ΔyabA)*, AK53 *(tetR-gfp/tetO^{-7} ΔyybE ΔyabA)*

Supplementary data references

1. Kobayashi, K., et al., *Essential Bacillus subtilis genes.* Proc Natl Acad Sci U S A, 2003. **100**(8): p. 4678-83.
2. Kunst, F., et al., *The complete genome sequence of the gram-positive bacterium Bacillus subtilis.* Nature, 1997. **390**(6657): p. 249-56.
3. Murray, H. and A. Koh, *Multiple regulatory systems coordinate DNA replication with cell growth in Bacillus subtilis.* PLoS Genet, 2014. **10**(10): p. e1004731.
4. Noirot-Gros, M.F., et al., *An expanded view of bacterial DNA replication.* Proc Natl Acad Sci U S A, 2002. **99**(12): p. 8342-7.
5. Claessen, D., et al., *Control of the cell elongation-division cycle by shuttling of PBP1 protein in Bacillus subtilis.* Mol Microbiol, 2008. **68**(4): p. 1029-46.
6. Le Breton, Y., N.P. Mohapatra, and W.G. Haldenwang, *In vivo random mutagenesis of Bacillus subtilis by use of TnYLB-1, a mariner-based transposon.* Appl Environ Microbiol, 2006. **72**(1): p. 327-33.
7. Lewis, P.J. and A.L. Marston, *GFP vectors for controlled expression and dual labelling of protein fusions in Bacillus subtilis.* Gene, 1999. **227**(1): p. 101-10.
